# Deep learning with leagues championship algorithm based intrusion detection on cybersecurity driven industrial IoT systems

**DOI:** 10.1038/s41598-025-15464-0

**Published:** 2025-08-19

**Authors:** Saud S. Alotaibi, Turki Ali Alghamdi

**Affiliations:** Department of Computer Science and Artificial Intelligence, College of Computing, Umm Al-Qura University, Makkah, Saudi Arabia

**Keywords:** Deep learning, Leagues championship algorithm, Cybersecurity, Internet of things, Feature selection, Computer science, Information technology

## Abstract

The Internet of Things (IoT) presents significant advantages to day-to-day life across a wide range of application domains, including healthcare automation, transportation, and smart environments. However, owing to the constraints of limited resources and computation abilities, IoT networks are subject to different cyber-attacks. Incorporating IDS into the cybersecurity-driven IIoT process contains cautious deployment, planning, and progressing management. Cybersecurity is crucial for the protection of sensitive data, safeguarding the privacy of users, and securing important substructures from malicious activities attempting unauthorized access or triggering interferences. Cyberattack detection performs a vital role in this defense scheme, employing advanced technologies like deep learning (DL) for analysing digital activities in real time. With the help of recognizing and responding to possible cyber-attacks quickly, cyberattack detection not only mitigates risks but reinforces the overall flexibility of the digital ecosystem against developing security challenges. This study presents a League Championship Algorithm Feature Selection with Optimal Deep Learning based Cyberattack Detection (CLAFS-ODLCD) technique for securing the digital ecosystem. The CLAFS-ODLCD technique focuses on the recognition and classification of cyberattacks in the IoT infrastructure. To achieve this, the CLAFS-ODLCD method utilizes the linear scaling normalization (LSN) approach for data pre-processing. Furthermore, the CLAFS-ODLCD method employs the CLAFS approach to choose optimal feature subset. Moreover, the detection and classification of the cyberattacks are accomplished by implementing the stacked sparse autoencoder (SSAE) approach. Finally, the hunger games search (HGS) optimizer is employed for optimum hyperparameter selection. The empirical analysis of the CLAFS-ODLCD method is examined under the WSN-DS dataset. The comparison study of the CLAFS-ODLCD method portrayed a superior accuracy value of 99.48% over existing models.

## Introduction

The IoT is a developing paradigm, which allows the relationship of computing capabilities and physical objects to link the Internet. The IoT can aid in to construction of efficient and flexible applications in numerous fields namely environmental monitoring, health care, and industrialized controlled processes^1^. While IoT can improve efficiency and productivity over intellectual and remote management, additionally it improves the cyber-attack risks. In most sectors, the IIoT can significantly raise operational efficiency, efficacy, and productivity. The IIoT will primarily improve current facilities and processes, then the ultimate goal is to generate unique and enormously improved goods and services. Various concerns identify how and where IIoT inventions and resolutions can lead to administrative changes, new and enhanced goods and services, and completely novel business models. The consideration of a digital ecosystem has a place on products or services about which partners, clients, and providers are gathered and interact^2^. The collaborations are sustained by a central digital environment and different ecosystem services thereby all companies will perform across businesses. These network impacts must be reinforced in whole industries and confirm more cooperation^3^. The advantages and connections to a digital ecosystem is individual and dependent upon the necessities of the system. Furthermore, industrial sector, ecosystems are progressively forming themselves along the business’s individual value chain. The attention here is on making a digital space for sharing, like transferring the data in real-time, and also capable of making business important decisions especially rapid and reliant on the evidence. Digital ecosystems are based on the sharing of data^4^. When implemented both utilization data and individual data is produced that should be protected. Data sovereignty and data security are then a main domain of digital ecosystems. The combination of extensive categories of users, all worked with customized access and IT systems, making a heterogeneous IT platform that provides various attack surfaces for cybercrime^5^. The IT security technique should be varied and complex as the method of operation of possible attackers. Both cyberattack crime and cyberattack terror have improved exponentially^6^. To help protect lives and ensure responsible use, it is recommended to establish ethical guidelines for the virtual world that align with real-world standards and values. Additionally, new security measures is needed to protect the private domain in the virtual world. This study presents a review of cyberattack detection. Cyberattacks are activities that effort to avoid security measures of computer systems^7^. Cyberattack detection is defined as “the difficulty of identifying the persons who will utilize a computer system without authorization and those who have authentic access to the system however, exploiting their privileges. Various techniques could be employed for attack detection that are widely considered into three main types anomaly-, hybrid-, and misuse-based detection. Misuse-based detection must be scanned by predetermined attack signatures and primarily employed for recognizing the known attacks^8^. This is beneficial to identify the known attacks with decreased false alarms rate (FAR). It needs a particular alteration of the signature and directions of attacks under the dataset. The anomaly-based method is proficient in recognizing both the attack categories such as unknown or known^9^. It will capture the network and host machine behavior and then find the anomalies as obtained from normal behavior. It is the major prevalent technique as it will identify the zero-day attacks. Several advantages of employing this approach, and among them is the modification of profiling activities because of which attackers become jumbled about which activities they follow to arrive and endure unidentified^10^. Apart from the advantages, there are also the limitations it assesses with higher FAR and the legitimate activities referred to as an anomaly in rare cases.

This study presents a League Championship Algorithm Feature Selection with Optimal Deep Learning based Cyberattack Detection (CLAFS-ODLCD) technique for securing the digital ecosystem. The CLAFS-ODLCD technique focuses on the recognition and classification of cyberattacks in the IoT infrastructure. To achieve this, the CLAFS-ODLCD method utilizes the linear scaling normalization (LSN) approach for data pre-processing. Furthermore, the CLAFS-ODLCD method employs the CLAFS approach to choose optimal feature subset. Moreover, the detection and classification of the cyberattacks are accomplished by implementing the stacked sparse autoencoder (SSAE) approach. Finally, the hunger games search (HGS) optimizer is employed for optimum hyperparameter selection. The empirical analysis of the CLAFS-ODLCD method is examined under the WSN-DS dataset. The major contribution of the CLAFS-ODLCD method is listed below.

- The CLAFS-ODLCD approach incorporates the LSN model for pre-processing the data effectually, thus ensuring that input features are scaled uniformly, which improves data quality, mitigates variability, and facilitates faster and more stable training, ultimately enhancing the overall performance and reliability of the cyberattack detection model.
- The CLAFS-ODLCD technique employs the CLAFS method to systematically choose the most relevant and informative features from the dataset, mitigating dimensionality and noise, which results in an enhanced model accuracy, faster computation, and enhanced generalization capability in detecting cyberattacks effectively.
- The CLAFS-ODLCD methodology utilizes the SSAE model for learning deep and meaningful representations from the input data, enabling robust detection and accurate classification of diverse cyberattacks by capturing intrinsic patterns and mitigating overfitting, thereby improving the overall efficiency and reliability of the security system.
- The CLAFS-ODLCD method implements the HGS technique for effectually fine-tuning its parameters, which optimizes overall performance by balancing exploration and exploitation during the search process, resulting in an enhanced detection accuracy and enhanced adaptability in dynamic network environments.
- The CLAFS-ODLCD model uniquely incorporates LSN, CLAFS, SSAE, and HGS into a unified framework, presenting a novel approach that effectually improves data preprocessing, feature selection, detection accuracy, and parameter optimization, resulting in an efficient and robust solution for accurate cyberattack detection and classification.

## Related works

Zainudin et al.^11^ established a lightweight protective and dependable blockchain-assisted federated learning (BFL)-based IDS technique. An authorized federated IDS was introduced based on the proof-of-authority (PoA) agreement. This developed model applied a hybrid client selection (HCS) method for choosing better metaverse edge devices. Moreover, an improved ERC-20 token-based incentive method is presented. In^12^, a BC system was presented. Similarly, a lightweight BC-based signature algorithm (LWBSA) technique is utilized. The notion’s resource limitations is alleviated through a central control to produce exchanged keys. The ELIB method is employed in three optimizations namely Lightweight agreement, the elliptic curve digital signature algorithm (ECDSA), and distributed throughput management (DTM). Malik et al.^13^ presented a secure platform by employing BC and DL-based techniques. Firstly, a BC leveraging method was designed by the bonobo optimizer method. Besides, the developed method presents the combination of Feistel architecture with optimum functions. Also, the deep reinforcement learning (DRL) technique is employed. Padmapriya and Srivenkatesh^14^ introduced a cryptography-assisted Multilevel Key Management with Enhanced K-Nearest Neighbor (CMLKM-EKNN) method, which produces the key sets in the IoT environment to make a powerful authentication. The EKNN method recognizes the adjacent nodes, assigns weights, and executes the analysis of the features in attack detection. The digital twin-based model to support increasing the cybersecurity of CPSs is developed. Haddad et al.^15^ introduced an AI-based security technique for the IoT infrastructure (AI-SM-IoT). This strategy was dependent upon the edge network of AI-assisted security modules for IoT emergency response. These introduced methods employed the idea of the cyberspace killing chain. Moreover, every challenge in the edge layer was distributed by incorporating AI security components into a distinct layer of AI-SM-IoT provided by services. Rajaee and Mazlumi^16^ examined an innovative robust technique, named as multi-agent distributed DL (MADDL) technique, the protective model with numerous distance relays is mapped into the multi-agent distributed model by applying the graph model. The DNN as a cyberattack detection model was supposed for all the agents. Therefore, the detection models were altered by exploiting trained data, attained by simulating the grid in diverse categories of errors. In^17^, the multi-step deep q learning network (MSDQN)-based DL method is proposed. The DL technique was implemented in the authentication procedure for identifying authenticated IoT devices and avoiding intermediary attacks among them. Alternatively, the MSDQN was connected to identify and lessen malware attacks and DDoS attacks in data transmission among diverse positions. Sikder et al.^18^ introduced empirical AI-based techniques. High confidence AE (HCAE), unsupervised learning method and temporal graph convolutional network (TGCN) with Attention, a supervised learning technique are the two 2 DL approaches. HCAE employs adapted hidden layers (HLs) for increasing the classification efficiency.

Nandanwar and Katarya^19^ proposed AttackNet, a DL-based security model that incorporates convolutional neural network (CNN) and gated recurrent unit (GRU) architectures to efficiently detect and classify botnet attacks in Industrial IoT (IIoT) environments. Devi, Nandal, and Sehrawat^20^ proposed a federated learning-based lightweight intrusion detection system (FL-LIDS) technique by using optimized DL models, comprising CNN and long short-term memory (LSTM), to detect Distributed Denial of Service (DDoS) attacks in resource-constrained wireless sensor networks (WSNs) while preserving data privacy in smart city environments. Wang et al.^21^ developed a two-layer network intrusion detection system (NIDS) using a CNN–bidirectional long short-term memory with attention (CNN-BiLSTM-Attention) methodology integrated with Stacking ensemble learning to improve detection of minority-class attacks. Khadidos et al.^22^ presented CyberSentry, a comprehensive security framework for supervisory control and data acquisition (SCADA) technique that integrates recursive multi-correlation-based information gain (RMIG) method for feature selection, Tri-Fusion Net for attack detection, and parrot-levy blend optimization (PLBO) approach for dynamic parameter tuning. Nandanwar and Katarya^23^ presented a Transfer learning-based cnn-bidirectional long short-term memory (TL-BILSTM) model for accurate detection and classification of Mirai and BASHLITE botnet attacks in IoT environments using real-time network traffic data. Nandanwar and Katarya^24^ introduced Cyber-Sentinet, a DL-based Intrusion Detection System (IDS) enhanced with Shapley Additive Explanations (SHAP), for accurate and interpretable cyberattack detection in cyber-physical systems within Industrial IoT environments. Kauhsik, Nandanwar, and Katarya^25^ proposed a novel solution using ML and DL models, based on four key research questions and a systematic literature review to improve data protection and device security. Nandanwar and Katarya^26^ provided a comprehensive overview of blockchain architecture, components, security challenges, and applications across domains like healthcare, IoT, smart grid, and defence, emphasizing its core principles and real-world relevance. Sattarpour, Barati, and Barati^27^ proposed EBIDS, an anomaly-based IDS using Bidirectional Encoder Representations from Transformers (BERT) technique, designed for efficient and accurate intrusion detection in resource-constrained IoT environments across network and application layers. Nandanwar and Katarya^28^ presented a BC-based decentralized application using Ethereum smart contracts, InterPlanetary File System, and Non-Interactive Zero-Knowledge Proof for ensuring secure, scalable, and private healthcare data management integrated with IoT and IDS.

Despite crucial improvements, existing models encounter challenges related to high computational complexity, limited scalability, and insufficient adaptability to dynamic IIoT environments. Various models depend heavily on centralized frameworks, which may introduce latency and single points of failure. Furthermore, the interpretability of DL-based IDS remains limited, mitigating trust in critical applications. IoT device constraints additionally restrict the deployment of complex models, resulting in suboptimal real-time detection. Moreover, few methods comprehensively address multi-layered security risks across heterogeneous IoT networks. The research gap is in developing lightweight, interpretable, and scalable IDS solutions capable of efficient detection under resource constraints while maintaining robustness against evolving cyber threats in diverse IIoT settings. Addressing this research gap requires innovative integration of optimized DL techniques with decentralized frameworks for improving security without compromising the performance or usability of the system.

## The proposed method

**Fig. 1:**
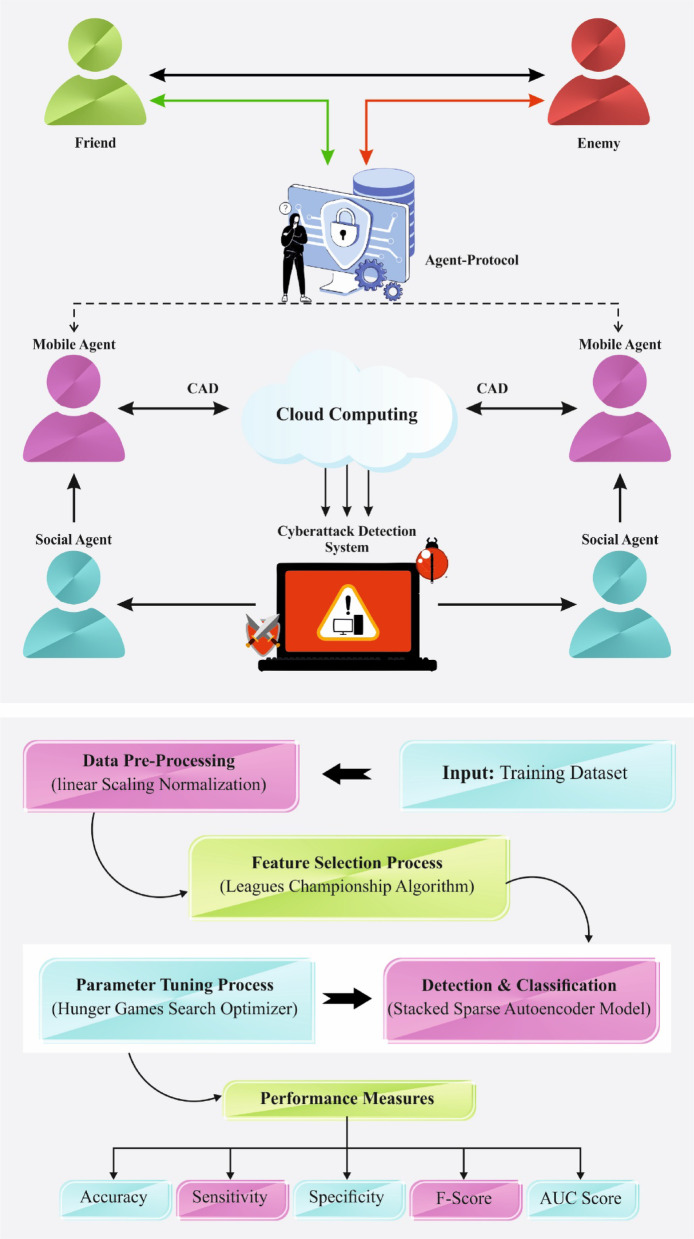
Working flow of CLAFS-ODLCD method.

In this work, a CLAFS-ODLCD model for securing the digital ecosystem is proposed. The CLAFS-ODLCD technique focuses on the recognition and classification of cyberattacks in the IoT infrastructure. To achieve this, the CLAFS-ODLCD technique involves various types of sub-processes namely LSN-based data normalization, CLA-based feature selection subset, SSAE-based classification, and HGS-based hyperparameter tuning. Figure 1 illustrates the working flow of the CLAFS-ODLCD technique.

### Data normalization

Initially, the CLAFS-ODLCD technique utilizes the LSN approach for data pre-processing. LSN is a data normalization method implemented in diverse domains, comprising ML and statistics for scaling and standardizing the numerical values within a particular range. Unlike some normalization techniques that target to center data about the mean or median, LSN linearly scales the input data to a predetermined interval at the range [0, 1] or [− 1, 1]. This procedure supports keeping the original correlation between data points when ensuring that the values are reliable and interpretable level. LSN is mainly beneficial in conditions wherein the absolute magnitude of data will not be important then, maintaining comparative variances is needed, providing more stable and efficient analyses in numerous applications.

### Feature selection using CLA

The CLAFS-ODLCD technique utilizes the CLAFS technique to choose an optimal subset of features. A new metaheuristic technique for solving continuous optimization problems, the CLA method was introduced by Kashan^29^. The team (each person) in the swarm of $L$ teams (leagues) has the feasible solution to the problem with $n$ players equivalent to the amount of variables. Team $i$ takes playing strength respective to the fitness rate following the construction of fake weekly league schedules. Based on this, the club plays together in pairs for $S\times \left(L-1\right)$ week where $t$ denotes the week and $S$ indicates the number of seasons. Playing outcomes define who wins and who loses. Based on the outcome of prior weeks, all sides form a new team match to prepare for the upcoming match. Under the direction of team formation, the configuration of the best team is selected with better playing strength and replaced by effective team formation.

#### League Schedule’s generation

The initial phase is to prepare the schedule that involves games for all the seasons. Each team plays together once the season under the round‐robin schedule. $L\left(L-1\right)/2$ competition exists, and $L$ must be the even integer. Then, the competition goes on for $S$ seasons. The CLA constructs an 8‐team $(L=8)$ sports league.

#### Evaluating winner or loser

Based on the standard playing strength, a winner and loser is selected, with playing strength $f\left({X}_{i}^{t}\right)$ and $f\left({X}_{J}^{t}\right)$ and formation ${X}_{i}^{t}=\left({x}_{i1}^{t},{x}_{\iota 2}^{t}, \dots ,{x}_{in}^{t}\right),{ X}_{i}^{t}$, correspondingly, ${i}\text{th}$ and ${j}\text{th}$ teams participating at ${t}\text{th}$ weeks are considered. ${p}_{i}^{t}$ represents the probability that ${i}\text{th}$ team will outdo ${j}\text{th}$ team in ${t}\text{th}$ week.

1 $${p}_{i}^{t}=\frac{f({X}_{j}^{t})-\widehat{f}}{f\left({X}_{j}^{t}\right)+f\left({X}_{i}^{t}\right)-2\widehat{f}}$$

In Eq. (1), the best team global team formation is denoted as $\widehat{f}$. Also, The probability that ${j}\text{th}$ teams can overcome ${i}\text{th}$ team is simultaneously defined by the random numbers within $[\text{0,1}$). If the number is greater than ${p}_{i}^{t}$ $i$ loses and $j$ wins. if the outcome is lesser than or equivalent to ${p}_{i}^{t}$ then team $i$ wins, and team $j$ loses.

#### New team formation

Based on the league schedule, the club that played with $l$ teams in ${t}\text{th}$ weeks, with ${i}\text{th}$ teams in week $t+1$, and, with ${i}\text{th}$ teams in ${t}\text{th}$ weeks, correspondingly, are represented by $i:l,j$, and $k$. Assume ${B}_{k}^{t},{ B}_{j}^{t}$, and ${B}_{i}^{t}=\left({b}_{i1}^{t}, {b}_{i2}^{t}, \dots ,{ b}_{in}^{t}\right)$ as the best team configuration for $k, i$, and $i$ teams at ${t}\text{th}$ weeks, correspondingly. It can affirm that for ${k}\text{th}$ teams to overcome ${l}\text{th}$ teams, ${i}\text{th}$ teams should come up with a playing style akin to that employed by ${k}\text{th}$ teams at ${t}\text{th}$ weeks, based on the strength of ${k}\text{th}$ teams, which $\left({B}_{k}^{t}-{B}_{i}^{t}\right)$ represents the gap vector amongst the playing strategy of ${k}\text{th}$ and ${i}\text{th}$ teams. Likewise, it steers clear of adopting the playing strategy that is analogous to ${k}\text{th}$ teams while concentrating on the deficiency of the team $({B}_{i}^{t}-{B}_{k}^{t})$. The information of the gap vector is integrated with constant parameters,${\psi }_{2}$‐approach, and ${\psi }_{1}$‐retreat, for generating a new team. The approach parameter is employed once $i$ team desires to go toward the rival. In contrast, the retreat parameter is employed if $i$ team distances itself from the competitor.

The swarm‐based technique was used to accomplish a globally optimal solution. The CLA can easily get stuck in local optimal solutions despite its effectiveness and simplicity, resulting in an imbalance in local exploitation and global exploration.

In the CLA model, the objective is combined into single objective thus weight finds the objective importance^30^.

2 $$Fitness\left(X\right)=\alpha \cdot E\left(X\right)+\beta *\left(1-\frac{\left|R\right|}{\left|N\right|}\right)$$

In Eq. (2), $Fitness(X)$ is the fitness value of $X$ subset$,$ $\alpha$, and $\beta$ are the weights of classifier error rate and the reduction ratio, $\alpha \in [\text{0,1}]$ and $\beta =(1-\alpha )$. $E(X)$ indicates the classifier error using the attributes selected in the $X$ subset, $|R|$ and $|N|$ are the amount of attributes selected and the amount of attributes in the original data correspondingly.

### Cyberattack detection using SSAE

**Fig. 2:**
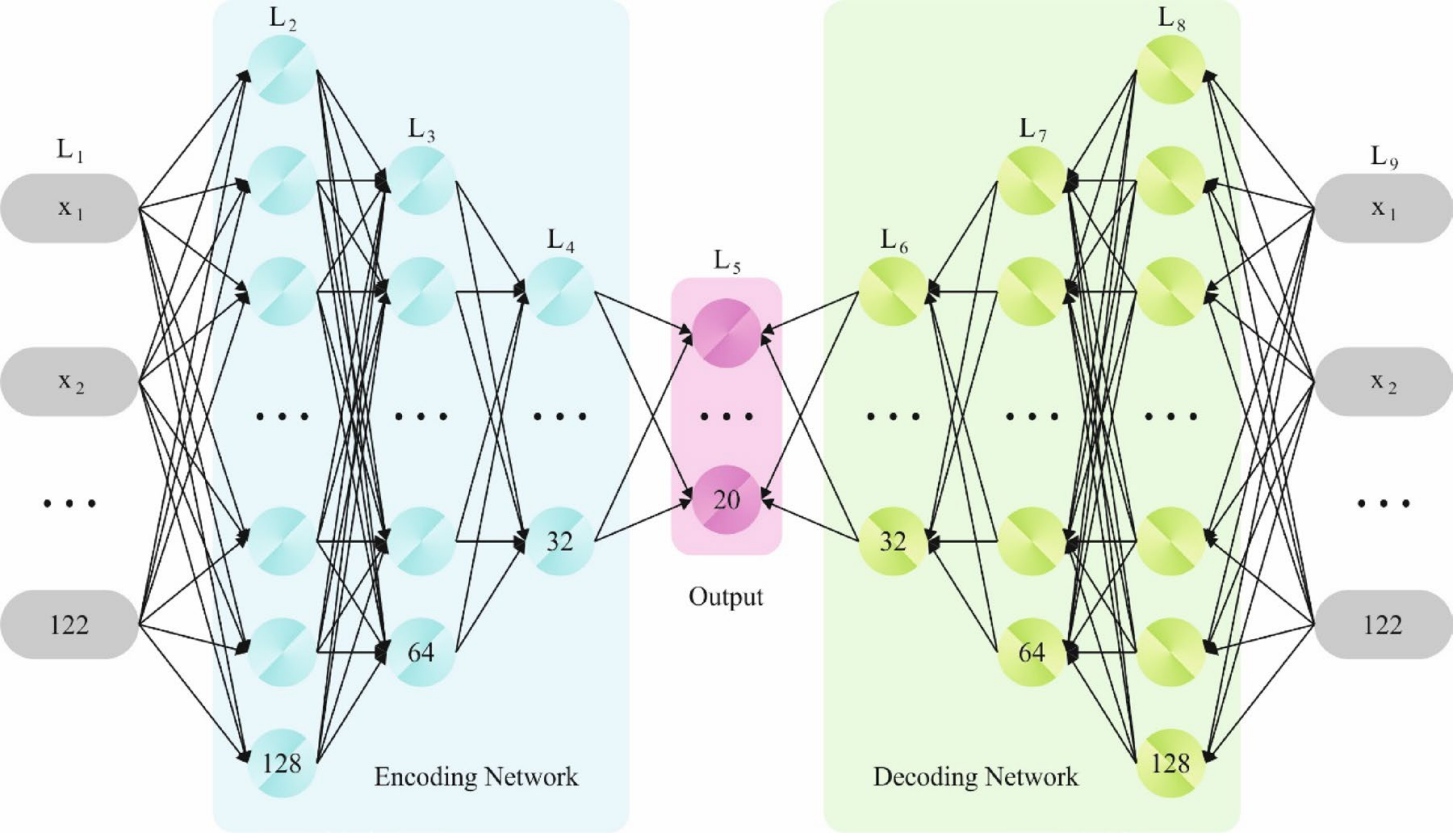
Architecture of SSAE.

In this phase, the detection and classification of the cyberattacks are performed by using the SSAE approach^31^. This model is chosen for its robust capability in learning deep hierarchical feature representations and detecting subtle and intrinsic attack patterns compared to conventional methods. This model also effectually mitigates noise and irrelevant data, thereby enhancing the detection accuracy and generalization. The technique also utilizes dropout regularization and early stopping strategies for addressing overfitting, ensuring the model does not memorize the training data but generalizes well to unseen samples. Furthermore, class imbalance is handled through techniques such as weighted loss functions or data augmentation, allowing SSAE to maintain robust performance across minority attack classes, which is significant for reliable intrusion detection in cybersecurity environments. Figure 2 illustrates the infrastructure of SSAE.

An autoencoder (AE) is an unsupervised learning method that mechanically absorbs the raw feature data and contains three layers namely HL, output, and input. The network of coding collects of input layer and an HL, and the decoder is made up of an output layer and an HL. The network of coding removes the original feature data.

$X=[{X}_{1},{ X}_{2}, \cdots ,{ X}_{n}{]}^{T}$ denotes the network input, and $n$ represents the amount of nodes, demonstrating the data size of the sample. The $h$ hidden features of the $X$ original data gained over the coding system are computed as below:

3 $$h=f\left(WX+b\right)$$

whereas $f$ specifies the Sigmoid activation function; $b$ and $W$ denote the biases and weights, correspondingly; the parameter $h$ is removed by coding; and $W$ size is $s\times n$, while $s$ represents the feature parameter size.

The decoder is employed to rebuild the original data of input, and the rebuilt data $Y$ is gained after decoding the $h$ hidden feature as below:

4 $$Y=U(W^{\prime}h+b^{\prime})$$

While, $Y=[{Y}_{1}, {Y}_{2}, \cdots , {Y}_{n}{]}^{T}$ represents the output data of the network; $U$ refers to the Sigmoid activation function; $b^{\prime}$ denotes the biases and $W^{\prime}$ represents the weights utilized in the coding stage. Whereas, $W{\prime}={W}^{T}.$

The AE employs stochastic gradient descent and backpropagation (BP) techniques to improve the set of parameter $\theta =\{W,b, W^{\prime}, b^{\prime}\}$ to diminish faults among data of input and output. Generally, the function of MSEis described as a loss function that is given below:

5 $${J}_{MSE}(\theta )=\frac{1}{m}\sum\limits_{i=1}^{m}\frac{1}{2}\| {X}^{\left(i\right)}-{Y}^{\left(i\right)}{\| }^{2}$$

Here, ${X}^{(i)}$ symbolizes the original data of the $i$ sample; ${Y}^{(i)}$ refers to the output data and $m$ denotes the total amount of training samples.

The SAE is created by inserting a term of sparse penalty to the $AE$ cost function. In the following equations, the sparse penalty term is definite:

6 $${J}_{spare}(\theta )=\beta \sum\limits_{j=1}^{s}KL(\rho \left.\Vert {\widehat{\rho }}_{j}\right)$$

7 $$KL(\rho \left.\Vert {\widehat{\rho }}_{j}\right)=\rho {\text{log}}_{2}\frac{\rho }{{\widehat{\rho }}_{j}}+\left(1-\rho \right)\text{log }\frac{1-\rho }{{1-\widehat{\rho }}_{j}}$$

8 $${\widehat{\rho }}_{j}=\frac{1}{m}\sum\limits_{i=1}^{m}({a}_{j}{X}^{(i)})$$

In Eq. (6), $\beta$ denotes the factor of sparse penalty that is employed to manage the weight in the loss function; ${\widehat{\rho }}_{j}$ represents the average activation value of HL; $s$ refers to the size of the HL; and $\rho$ specifies the parameter of sparse. Equation (7) denotes the calculation of relative entropy formulation, which is applied to determine the degree of deviance among the dual supplies. Equation (8) computes the average activation value of HL, whereas ${a}_{j}$ designates the amount of activity in the $j$ unit of the HL.

9 $$J\left(\theta \right)={J}_{MSE}\left(\theta \right)+{J}_{sparse}\left(\theta \right)$$

The above-mentioned formula is the SAE loss function. Where the 1st term denotes the function of MSE and the 2nd term refers to the sparse penalty term.

### HGS-based hyperparameter tuning

Finally, the HGS optimizer is employed for the optimum hyperparameter selection. HGS is a population-reliant optimizer model that has resolved restricted and free issues while maintaining the feature^32^. The sub-sections define the numerous steps in an algorithm of HGS.

#### Moving near food

Thus the below-mentioned mathematical formulations are formed to pretend the reduction mode and imitate its future behavior.

10 $$\overrightarrow{Y(t+1)}=\left\{\begin{array}{l}\overrightarrow{Y(t)}\cdot \left(1+\mathfrak{R}m\left(1\right)\right), {\mathfrak{R}}_{1}<k\\ \overrightarrow{{Z}_{1}}\cdot \overrightarrow{{Y}_{a}}-\overrightarrow{S}\cdot \overrightarrow{{Z}_{2}}\left|\overrightarrow{{Y}_{a}}-\overrightarrow{Y\left(t\right)}\right|, {\mathfrak{R}}_{1}>k,{\mathfrak{R}}_{2}>F\\ \overrightarrow{{Z}_{1}}\cdot \overrightarrow{{Y}_{a}}+\overrightarrow{S}\cdot \overrightarrow{{Z}_{2}}.\left|\overrightarrow{{Y}_{a}}-\overrightarrow{Y\left(t\right)}\right|, {\mathfrak{R}}_{1},{\mathfrak{R}}_{2}<F>k,\end{array}\right.$$

whereas, $\overrightarrow{S}$ denotes the ranges among $-b$, and $b$. The randomly generated numbers in the range $[0\, \text{and}\, 1]$ are signified as ${\mathfrak{R}}_{1}$ and ${\mathfrak{R}}_{2}$. The existing iteration is represented as $t$. $\mathfrak{R}m(1)$ is a normal distribution of random numbers. $\overrightarrow{{Z}_{1}}$ and $\overrightarrow{{Z}_{2}}$ are the hunger’s weight. Individuals’ full position is reflected by utilizing the $\overrightarrow{Y\left(t\right)}$ and the initial location is $k$. $\overrightarrow{{Y}_{a}}$ is represented by the position of a random individual. The below-given expression is for originating F.

11 $$F=sech\left(\left|E\left(j\right)-Bes{t}_{fitness}\right|\right)$$

whereas, $j\in \text{1,2},\ldots ,m$. $E\left(j\right)$ denotes the fitness value and $Bes{t}_{fitness}$ represents the optimum fitness attained in the existing iteration method. The hyperbolic function $\left(sech\left(y\right)=\frac{2}{{e}^{y}+{e}^{-y}}\right)$ is denoted as $such$. The calculation for $\overrightarrow{S}$ is set below:

12 $$\overrightarrow{S}=2\times b\times \mathfrak{R}-b$$

13 $$b=2\times \left(1-\frac{t}{\text{ maximum}_{iteration}}\right)$$

Here, $\mathfrak{R}$ symbolizes the random integer within $[\text{0,1}]$. The biggest number in an iteration is represented by $\text{maximum}_{iteration}.$

#### Hunger role

The starvation features who are searching are demonstrated utilizing mathematical simulation. The formulation for $\overrightarrow{{Z}_{1}}$ is provided below:

14 $$\overrightarrow{{Z}_{1}(j)}=\left\{\begin{array}{l}hungry\left(j\right)\cdot \frac{M}{su{m}_{hungry}}\times {\mathfrak{R}}_{4}, {\mathfrak{R}}_{3}<k\\ 1\quad \quad\,\, {\mathfrak{R}}_{3}>k\end{array}\right.$$

The equation for $\overrightarrow{{Z}_{2}}$ is as follows:

15 $$\overrightarrow{{Z}_{2}(j)}=\left(1-exponential\left(-\left|hungry\left(j\right)-su{m}_{{h}_{ll}mgry}\right|\right)\right)\times {\mathfrak{R}}_{5}\times 2$$

Each individual’s starvation is signified by employing the variable $hungry (j)$. The individual’s amount is denoted by $M$. $su{m}_{hungry}$ is the sum of the entire individual’s hunger experiences. Random numbers among $0$ and 1 are denoted by ${\mathfrak{R}}_{3}$, ${\mathfrak{R}}_{4}$, and ${\mathfrak{R}}_{5}$. The $hungry (j)$ representation is the resultant utilizing Eq. (16).

16 $$hungry\left( j \right) = \left\{ {\begin{array}{*{20}l} {0,} \hfill & {OF~\left( j \right) = Bestfitness} \hfill \\ {ungry\left( j \right) + hunger_{{sensation}} ,} \hfill & {h~OF\left( j \right) = Bestfitness} \hfill \\ \end{array} } \right.$$

In the existing iteration, all individual fitness is kept by $OF (j)$. The calculation for $hunge{r}_{sensation}$ is mentioned as follows:

17 $$\begin{aligned} hunger_{threshold} & = \frac{E\left( j \right) - bestfitness}{{worstfitness - bestfitness}} \times {\Re }_{6} \times 2 \\ & \quad \times \left( {upper_{bound} - lower_{bound} } \right) \\ \end{aligned}$$

18 $$hunger_{sensation} = \left\{ {\begin{array}{*{20}l} {lower_{bound} \times \left( {1 + {\Re }} \right){ },} \hfill & {hunger_{thershold} < lower_{bound} } \hfill \\ {hunger_{thershold} ,} \hfill & {hunger_{thershold} \ge lower_{bound} } \hfill \\ \end{array} } \right.$$

whereas, ${\mathfrak{R}}_{6}$ is signified by the random number between 0 and 1. The hunger threshold is symbolized by the $hunge{r}_{threshold}$. The fitness value of all individuals is represented by $E(j)$. The worst and best fitness achieved throughout the present procedure of iterations is denoted by $wors{t}_{fitness}$ and $bes{t}_{fitness}$. The $lowe{r}_{bound}$ and $uppe{r}_{bound}$ are denoted by the lower and upper boundaries of the problem. There is a lower boundary $(lowe{r}_{bound})$, to the feeling of hunger $\left(hunge{r}_{sensation}\right)$.

The fitness selection was the major factor that affected the performance of the HGS methodology. The hyperparameter selection method comprises the solution encoder process to estimate the efficacy of candidate solutions. Here, the HGS technique estimates precision as the key criterion for designing the FF.

19 $$Fitness =\text{ max }\left(P\right)$$

20 $$P=\frac{TP}{TP+FP}$$

where $TP$ and $FP$ are the true and the false positive values.

## Result analysis

The simulation validation of the CLAFS-ODLCD technique is examined under the WSN-DS dataset^33^. The method runs on Python 3.6.5 with an i5-8600k CPU, 4GB GPU, 16GB RAM, 250GB SSD, and 1TB HDD, using a 0.01 learning rate, ReLU, 50 epochs, 0.5 dropout, and batch size 5. It comprises 374,661 samples with 5 classes as shown in Table 1.

**Table 1:** Details of the dataset.

Classes	No. of Samples
Normal	340,066
Blackhole	10,049
Grayhole	14,596
Flooding	3312
Scheduling Attacks	6638
Total No. of Samples	374,661

**Fig. 3:**
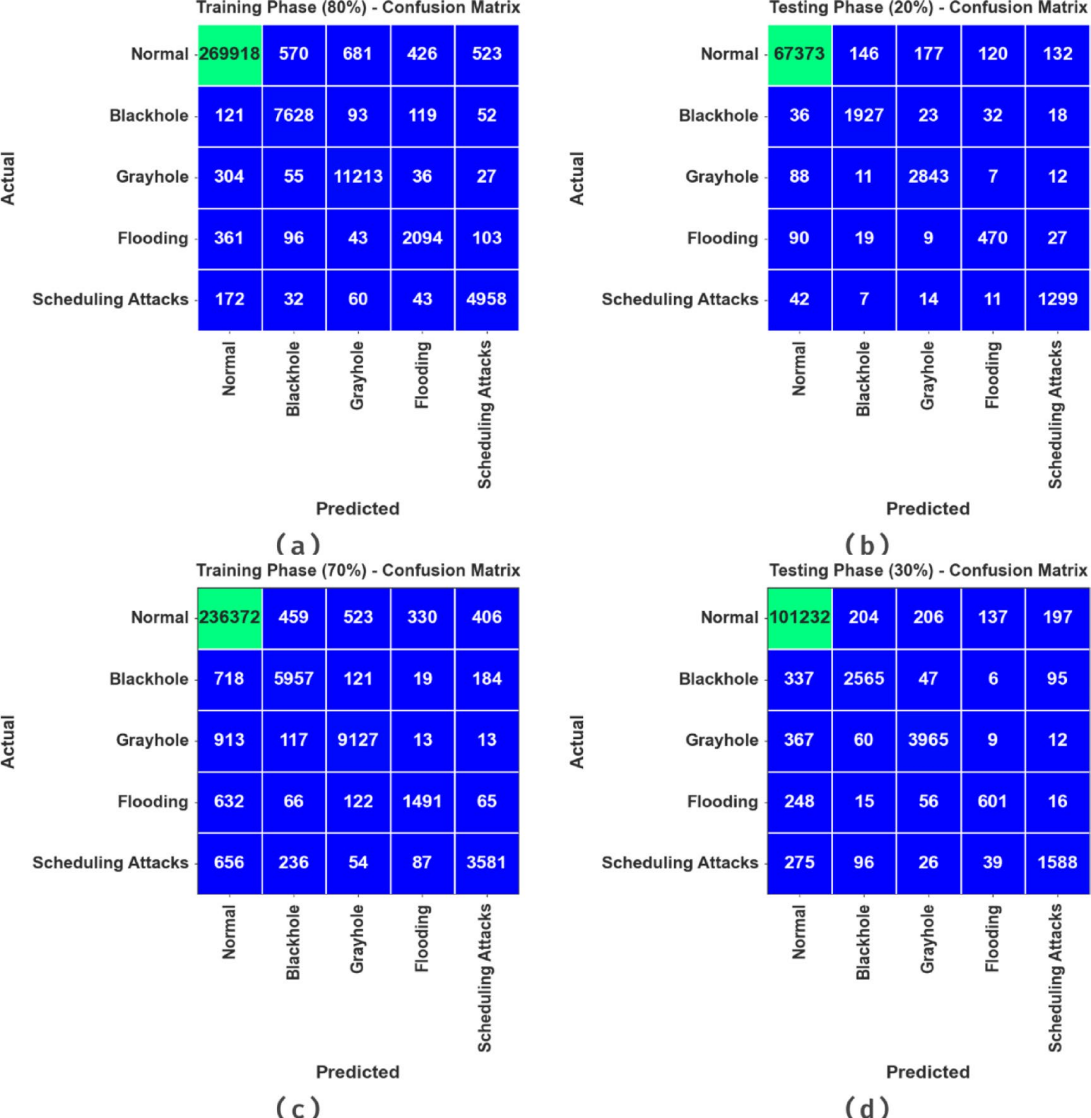
Confusion matrices of** a**,** b** 80%TRAPH:20%TESPH and** c**,** d** 70%TRAPH:30%TESPH.

The confusion matrices made by the CLAFS-ODLCD technique on 80%TRAPH:20%TESPH and 70%TRAPH:30%TESPH are demonstrated in Fig. 3. The results indicate effective detection with all five classes.

**Fig. 4:**
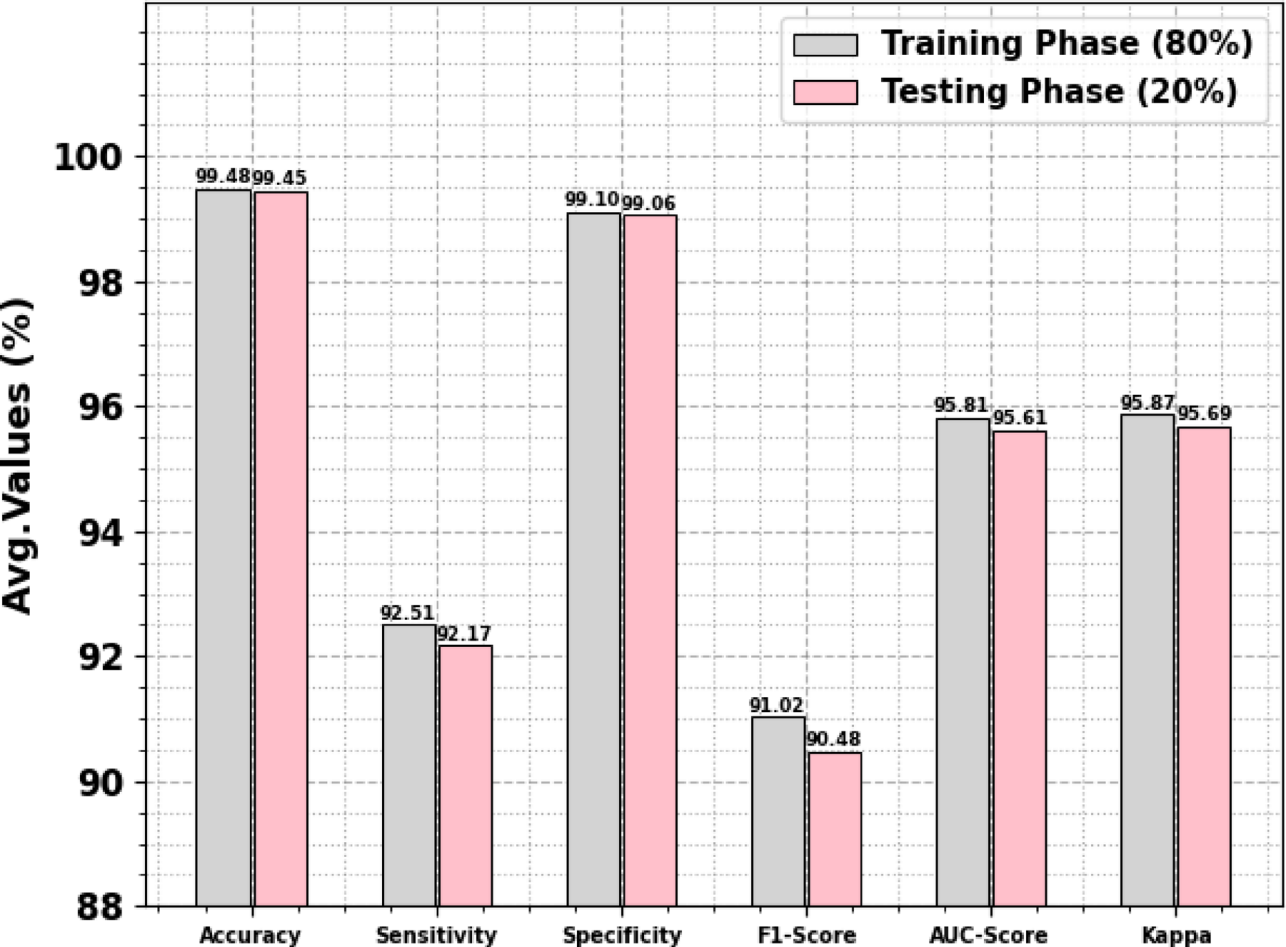
Average of the CLAFS-ODLCD model on 80%TRAPH and 20%TESPH.

Table 2 and Fig. 4. reveals the attack recognition results of the CLAFS-ODLCD approach are reported under 80%TRAPH and 20%TESPH. The experimental outcomes reported that the CLAFS-ODLCD approach gains effectual performance with distinct classes. With 80%TRAPH, the CLAFS-ODLCD technique attains an average $acc{u}_{y}$ of 99.48%, $sen{s}_{y}$ of 92.51%, $spe{c}_{y}$ of 99.10%, ${F}_{score}$ of 91.02%, $AU{C}_{score}$ of 95.81%, and Kappa of 95.87%. Furthermore, depending on 20%TESPH, the CLAFS-ODLCD technique obtains an average $acc{u}_{y}$ of 99.45%, $sen{s}_{y}$ of 92.17%, $spe{c}_{y}$ of 99.06%, ${F}_{score}$ of 90.48%, $AU{C}_{score}$ of 95.61%, and Kappa of 95.69%, correspondingly.

**Table 2:** Attack detection results of the CLAFS-ODLCD model under 80%TRAPH and 20%TESPH.

Classes	$Acc{u}_{y}$	$Sen{s}_{y}$	$Spe{c}_{y}$	${F}_{score}$	$AU{C}_{score}$	Kappa
TRAPH (80%)						
Normal	98.95	99.19	96.53	99.42	97.86	97.92
Blackhole	99.62	95.20	99.74	93.06	97.47	97.54
Grayhole	99.57	96.37	99.70	94.52	98.03	98.09
Flooding	99.59	77.64	99.79	77.34	88.72	88.80
Scheduling Attacks	99.66	94.17	99.76	90.74	96.96	97.02
Average	99.48	92.51	99.10	91.02	95.81	95.87
TESPH (20%)						
Normal	98.89	99.15	96.34	99.39	97.74	97.81
Blackhole	99.61	94.65	99.75	92.96	97.20	97.27
Grayhole	99.54	96.01	99.69	94.34	97.85	97.93
Flooding	99.58	76.42	99.77	74.90	88.10	88.17
Scheduling Attacks	99.65	94.61	99.74	90.81	97.18	97.26
Average	99.45	92.17	99.06	90.48	95.61	95.69

**Fig. 5:**
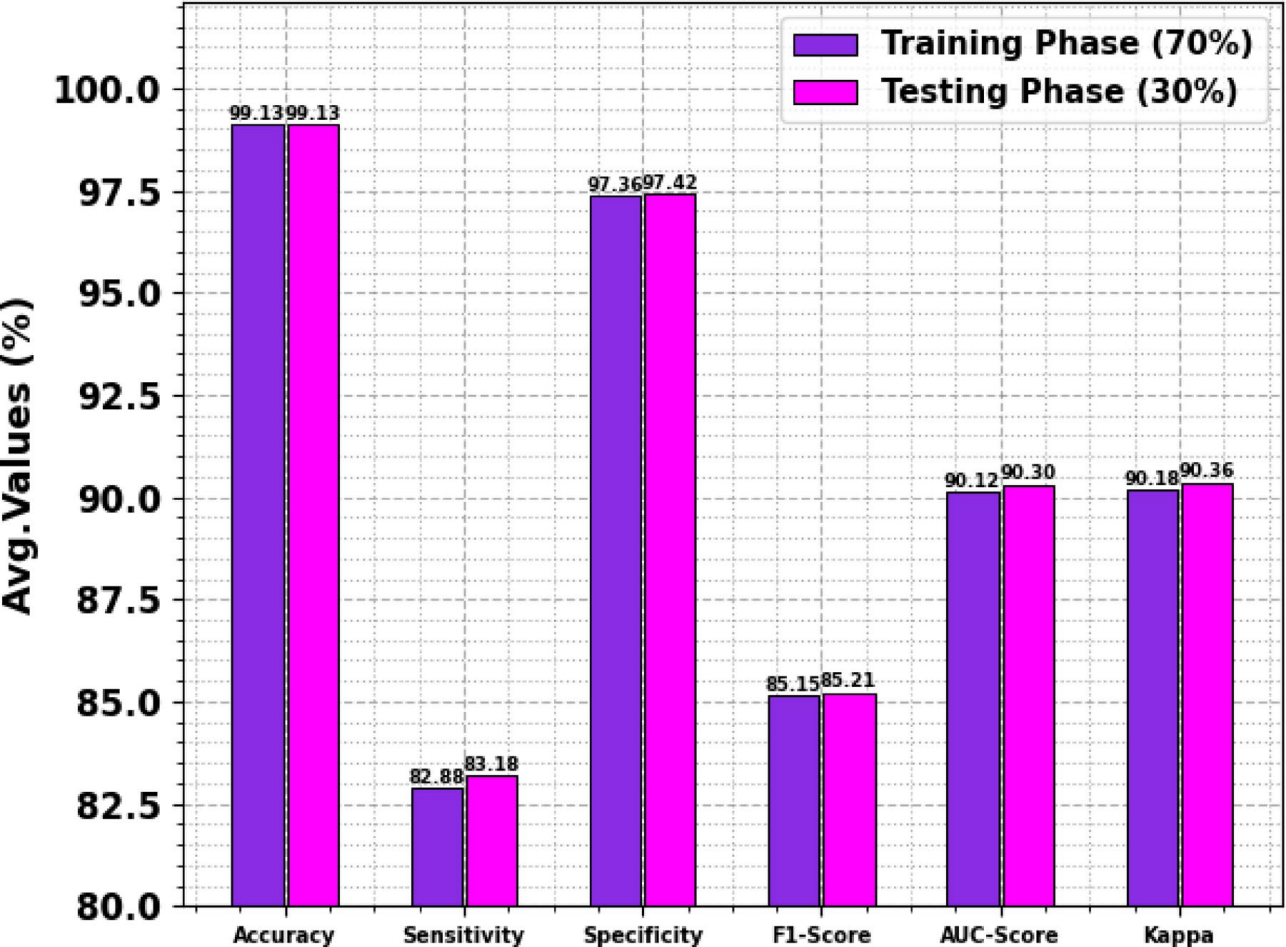
Average of the CLAFS-ODLCD method under 70%TRAPH and 30%TESPH.

Table 3 and Fig. 5 illustrates the attack detection results of the CLAFS-ODLCD method are reported under 70%TRAPH and 30%TESPH. The experimental outcomes stated that the CLAFS-ODLCD technique gains effectual performance with distinct classes.

**Table 3:** Attack detection outcomes of the CLAFS-ODLCD method at 70%TRAPH and 30%TESPH.

Classes	$Acc{u}_{y}$	$Sen{s}_{y}$	$Spe{c}_{y}$	${F}_{score}$	$AU{C}_{score}$	Kappa
TRAPH (70%)						
Normal	98.23	99.28	87.92	99.03	93.60	93.67
Blackhole	99.27	85.11	99.66	86.12	92.38	92.44
Grayhole	99.28	89.63	99.67	90.68	94.65	94.71
Flooding	99.49	62.75	99.83	69.09	81.29	81.35
Scheduling Attacks	99.35	77.61	99.74	80.81	88.68	88.73
Average	99.13	82.88	97.36	85.15	90.12	90.18
TESPH (30%)						
Normal	98.25	99.27	88.23	99.04	93.75	93.80
Blackhole	99.23	84.10	99.66	85.64	91.88	91.95
Grayhole	99.30	89.85	99.69	91.01	94.77	94.82
Flooding	99.53	64.21	99.83	69.56	82.02	82.09
Scheduling Attacks	99.33	78.46	99.71	80.77	89.08	89.13
Average	99.13	83.18	97.42	85.21	90.30	90.36

With 70%TRAPH, the CLAFS-ODLCD methodology reaches an average $acc{u}_{y}$ of 99.13%, $sen{s}_{y}$ of 82.88%, $spe{c}_{y}$ of 97.36%, ${F}_{score}$ of 85.15%, $AU{C}_{score}$ of 90.12%, and Kappa of 90.18%, appropriately. Furthermore, depending on 30%TESPH, the CLAFS-ODLCD model achieves an average $acc{u}_{y}$ of 99.13%, $sen{s}_{y}$ of 83.18%, $spe{c}_{y}$ of 97.42%, ${F}_{score}$ of 85.21%, $AU{C}_{score}$ of 90.30%, and Kappa of 90.36%, correspondingly.

**Fig. 6:**
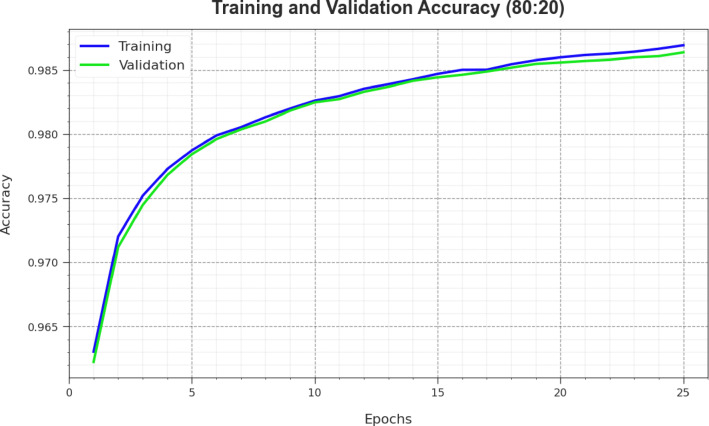
$Acc{u}_{y}$ curve of the CLAFS-ODLCD method at 80%TRAPH and 20%TESPH.

The performance of the CLAFS-ODLCD technique on 80%TRAPH and 20%TESPH is graphically shown in Fig. 6 for training accuracy (TRAC) and validation accuracy (VLAC) curves. The experimental result demonstrates the meaningful insight into the behavior of the CLAFS-ODLCD technique across numerous epochs, indicating its learning method and generalisabilities. Particularly, this figure points out a consistent development in the TRAC and VLAC with increasing epochs. It also guarantees the flexible nature of the CLAFS-ODLCD method in the pattern recognition technique on TR and TS datasets. The increasing tendency in VLAC describes the capacity of the CLAFS-ODLCD method to adjust to the TR dataset and also excels in presenting the correct classification of unseen datasets, representing strong generalization.

**Fig. 7:**
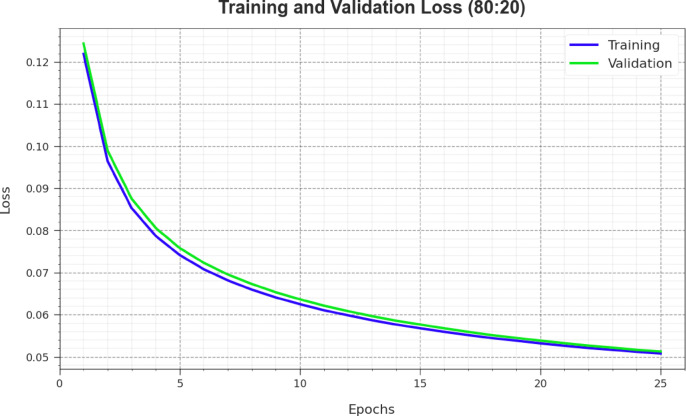
Loss curve of the CLAFS-ODLCD approach under 80%TRAPH and 20%TESPH.

Figure 7 demonstrates a comprehensive review of the training loss (TRLOS) and validation loss (VALOS) outcomes of the CLAFS-ODLCD technique on 80%TRAPH and 20%TESPH over different epochs. The continuous decrease in TRLOS underlines the CLAFS-ODLCD approach improving the weights and decreasing the classifier error on both datasets. The experimental result illustrates a clear knowledge of the CLAFS-ODLCD model’s relationship with the TR data, which emphasizes its ability to capture patterns inside both datasets. Particularly, the CLAFS-ODLCD approach continuously improves its parameters in minimizing the changes among the real and prediction TR classes.

**Fig. 8:**
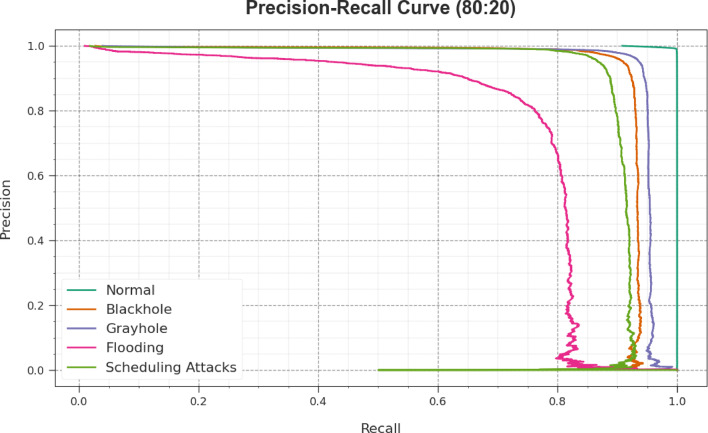
PR curve of the CLAFS-ODLCD approach under 80%TRAPH and 20%TESPH.

Inspecting the PR curve, as shown in Fig. 8, the outcome proved that the CLAFS-ODLCD approach on 80%TRAPH and 20%TESPH progressively obtained better PR values through all the classes. It proves the better abilities of the CLAFS-ODLCD model in the recognition of diverse classes, which exhibit ability in the recognition of classes.

**Fig. 9:**
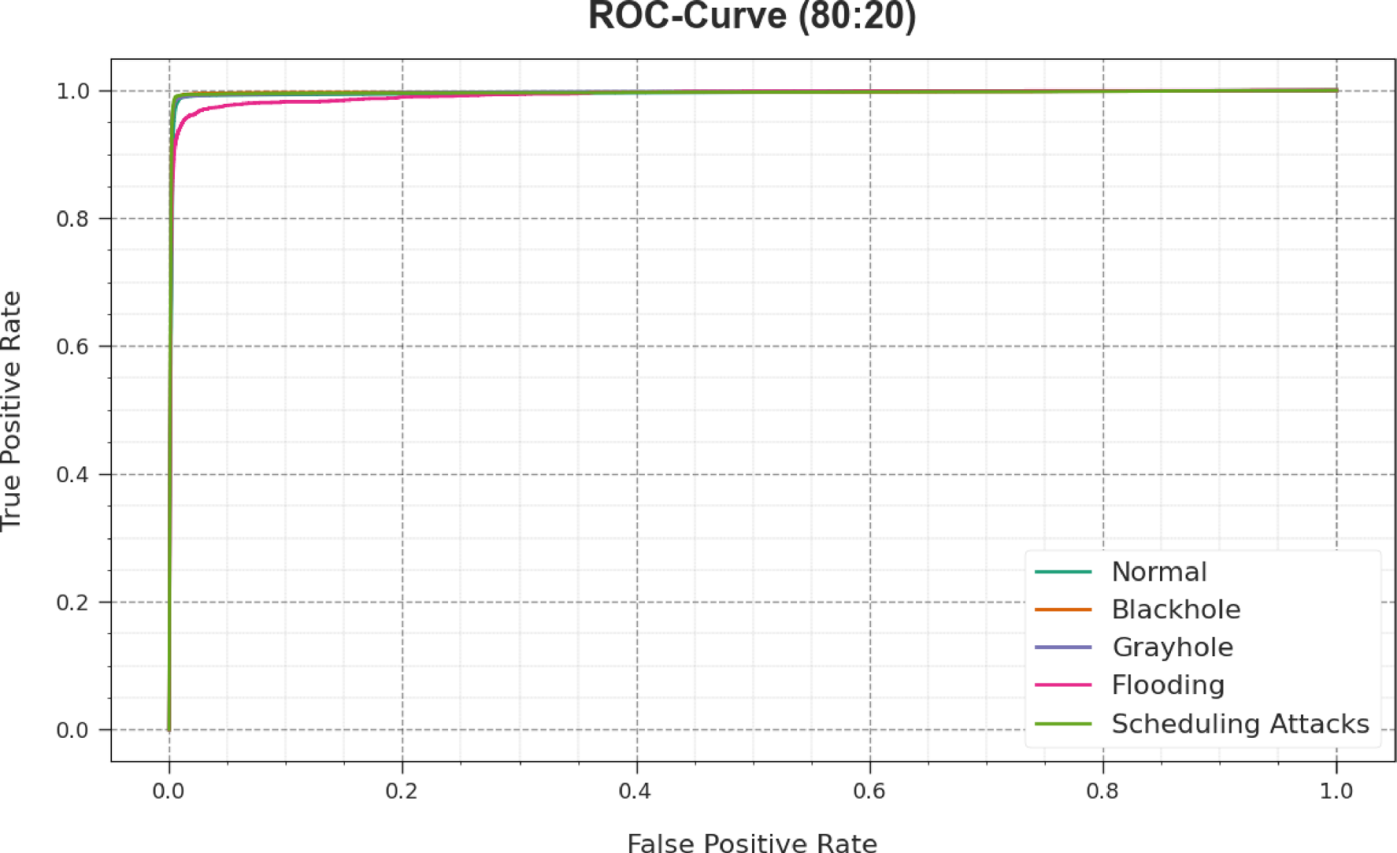
ROC curve of the CLAFS-ODLCD approach under 80%TRAPH and 20%TESPH.

Figure 9 depicts that ROC curves produced by the CLAFS-ODLCD technique on 80%TRAPH and 20%TESPH exceeded the classification of distinct labels. It presents a comprehensive understanding of the tradeoffs between FRP and TPR over various detection threshold values and epochs. The experimental results highlighted the superior classifier outcome of the CLAFS-ODLCD methods on diverse classes, which outlines the efficiency in addressing various classifier problems.

**Fig. 10:**
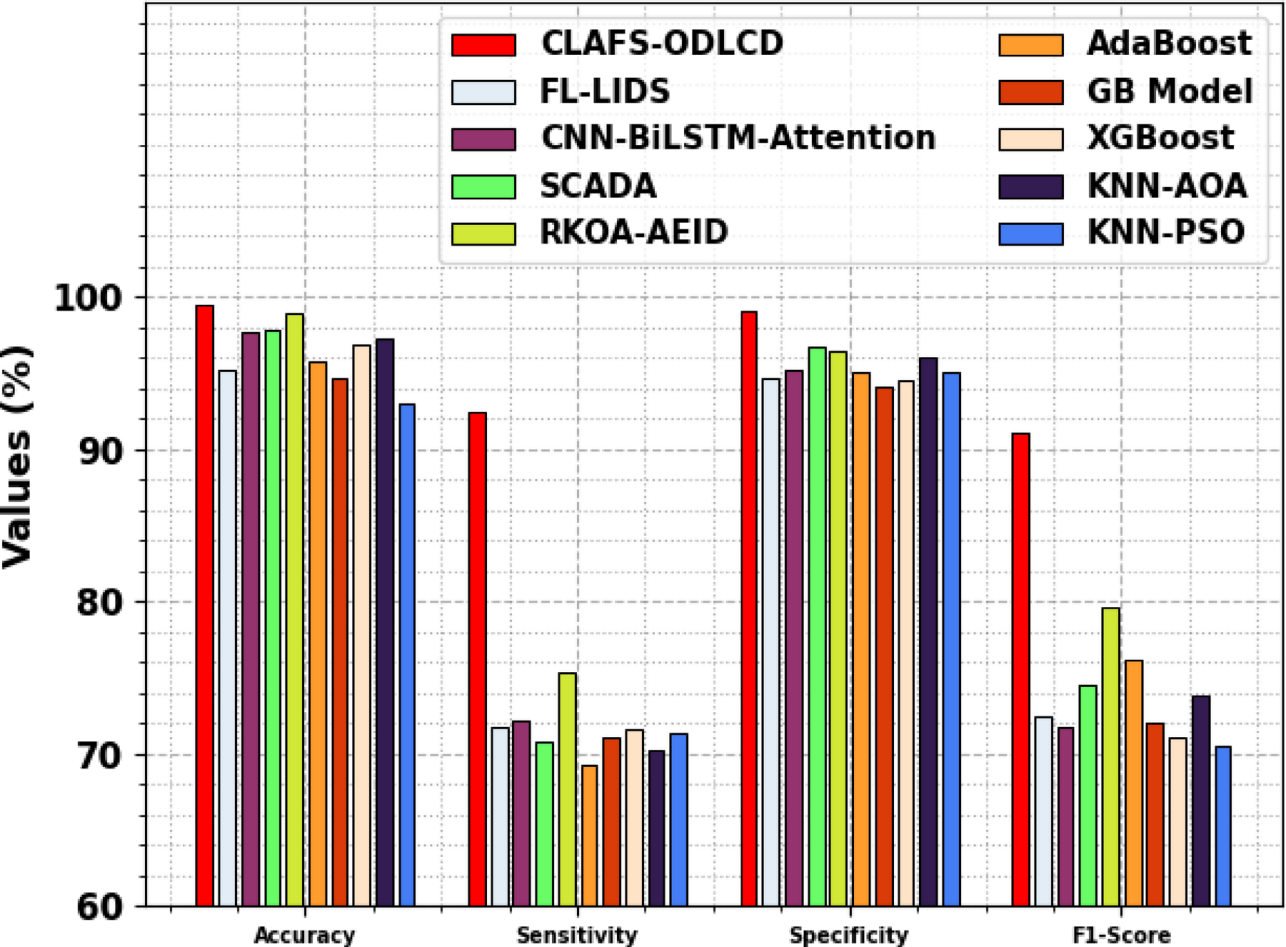
Comparative outcomes of the CLAFS-ODLCD model with existing techniques.

The performance of the CLAFS-ODLCD technique is compared with existing models in Table 4 and Fig. 10^20–22,34^. The result demonstrates that the CLAFS-ODLCD method gains effectual performance. It is noticed that the KNN-PSO, GB, and AdaBoost models highlighted ineffectual performance. Likewise, the FL-LIDS, CNN-BiLSTM-Attention, SCADA models attained slightly lower results. Simultaneously, the KNN-AOA and XGBoost models have resulted in moderately improved results. Although the RKOA-AEID technique has gained reasonable performance, the CLAFS-ODLCD method reaches greater performance with a maximal $acc{u}_{y}$ of 99.48%, $sen{s}_{y}$ of 92.51%, $spe{c}_{y}$ of 99.10%, and ${F}_{score}$ of 91.02%.

**Table 4:** Comparative outcomes of the CLAFS-ODLCD model with existing techniques^20–22,34^.

Methods	$Acc{u}_{y}$	$Sen{s}_{y}$	$Spe{c}_{y}$	${F}_{score}$
CLAFS-ODLCD	99.48	92.51	99.10	91.02
FL-LIDS	95.29	71.82	94.74	72.49
CNN-BiLSTM-Attention	97.66	72.22	95.25	71.75
SCADA	97.90	70.78	96.74	74.51
RKOA-AEID	99.00	75.39	96.52	79.57
AdaBoost	95.75	69.27	95.07	76.20
GB Model	94.66	71.10	94.15	71.98
XGBoost	96.89	71.57	94.49	71.06
KNN-AOA	97.28	70.23	96.10	73.90
KNN-PSO	92.96	71.36	95.15	70.54

**Fig. 11:**
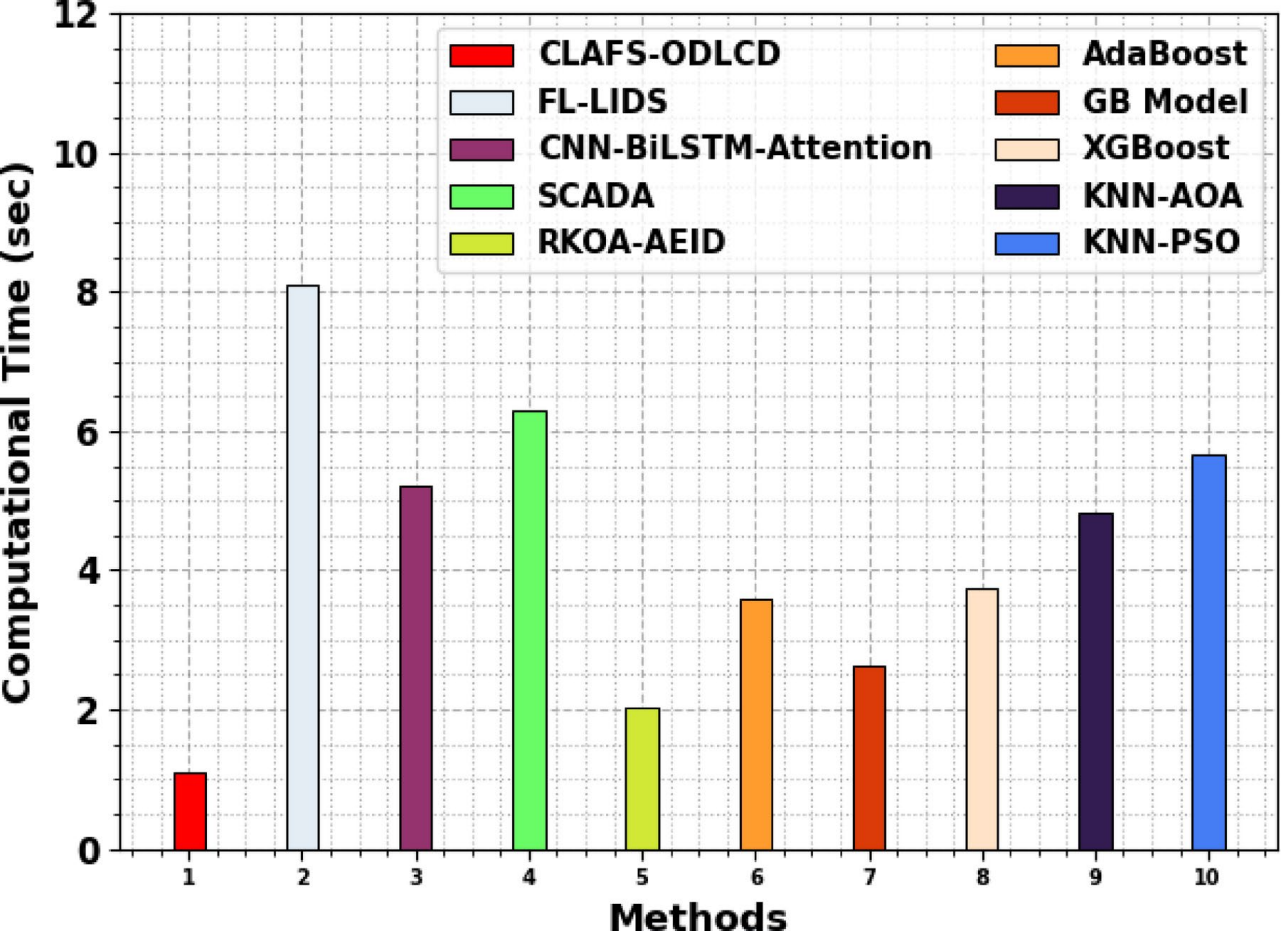
CT analysis of the CLAFS-ODLCD methodology with existing models.

An extensive computational time (CT) outcome of the CLAFS-ODLCD technique is compared with existing approaches in Table 5 and Fig. 11. The CLAFS-ODLCD technique is highly efficient, completing tasks in just 1.09 s, which is significantly faster than other techniques. For instance, FL-LIDS requires 8.09 s, CNN-BiLSTM-Attention takes 5.22 s, and SCADA completes in 6.31 s. Other models like RKOA-AEID, AdaBoost, GB Model, and XGBoost have CTs of 2.04, 3.60, 2.64, and 3.75 s, respectively, while KNN variants KNN-AOA and KNN-PSO require 4.84 and 5.66 s. This highlights that the CLAFS-ODLCD model reduces computational overhead by approximately 70 to 85 percent compared to these advanced models, emphasizing its suitability for real-time and resource-constrained environments.

**Table 5:** CT outcome of the CLAFS-ODLCD methodology with existing models.

Methods	CT (sec)
CLAFS-ODLCD	1.09
FL-LIDS	8.09
CNN-BiLSTM-Attention	5.22
SCADA	6.31
RKOA-AEID	2.04
AdaBoost	3.60
GB Model	2.64
XGBoost	3.75
KNN-AOA	4.84
KNN-PSO	5.66

**Fig. 12:**
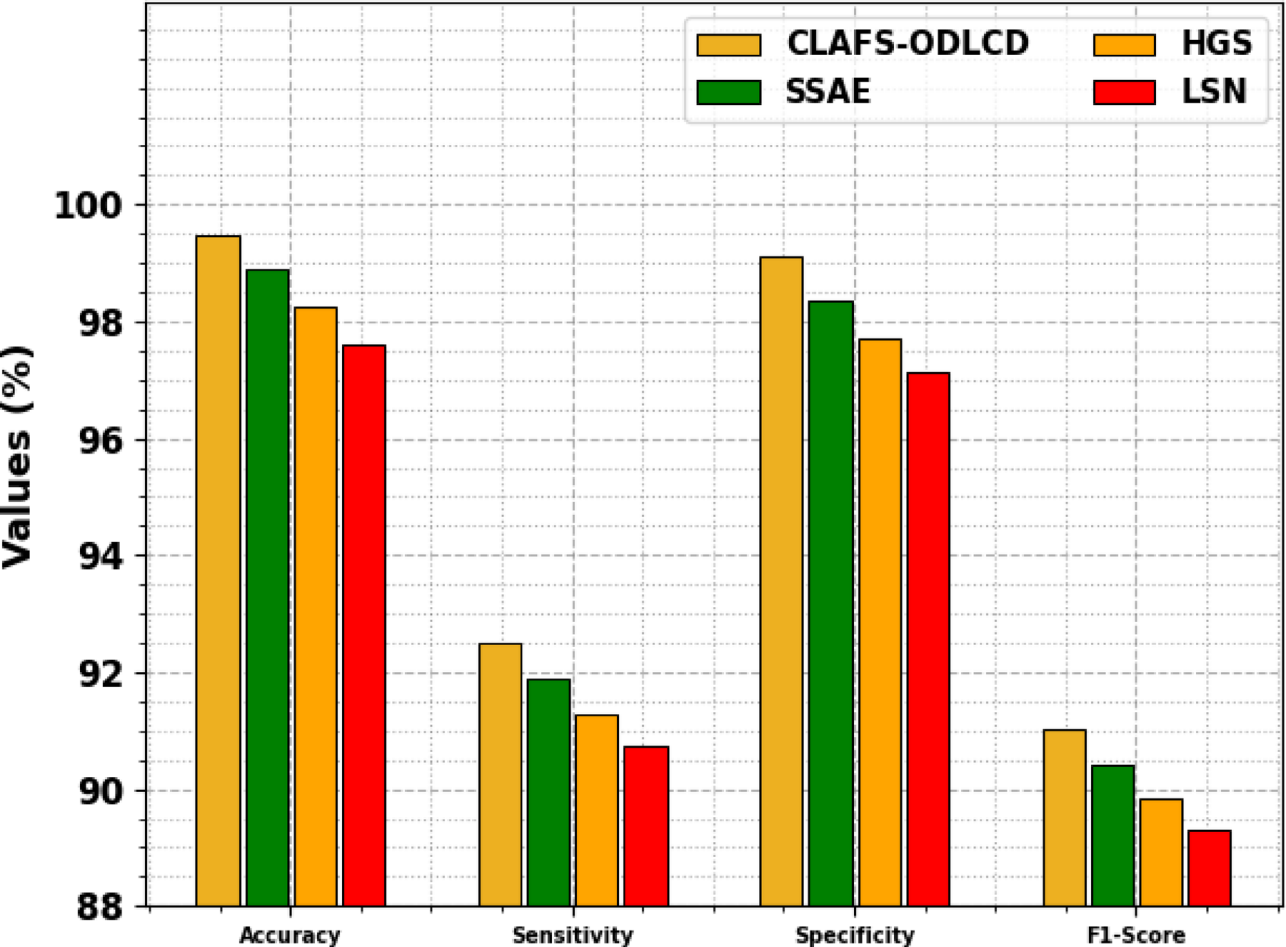
Ablation study results comparing CLAFS-ODLCD method with existing techniques.

Table 6 and Fig. 12 specifies the ablation study of the CLAFS-ODLCD approach. The ablation study results show that the CLAFS-ODLCD method achieves an $acc{u}_{y}$ of 99.48%, $sen{s}_{y}$ of 92.51%, $spe{c}_{y}$ of 99.10%, and an ${F}_{score}$ of 91.02%. SSAE records an $acc{u}_{y}$ of 98.91%, $sen{s}_{y}$ of 91.89%, $spe{c}_{y}$ of 98.35%, and ${F}_{score}$ of 90.43%. HGS achieves an accuracy of 98.26%, $sen{s}_{y}$ of 91.29%, $spe{c}_{y}$ of 97.70%, and ${F}_{score}$ of 89.85%. Lastly, LSN attains an $acc{u}_{y}$ of 97.61%, $sen{s}_{y}$ of 90.75%, $spe{c}_{y}$ of 97.12%, and an ${F}_{score}$ of 89.32%. These values illustrate the superior performance of the CLAFS-ODLCD method across all metrics.

**Table 6:** Ablation study results comparing CLAFS-ODLCD method with existing techniques.

Methods	$Acc{u}_{y}$	$Sen{s}_{y}$	$Spe{c}_{y}$	${F}_{score}$
CLAFS-ODLCD	99.48	92.51	99.10	91.02
SSAE	98.91	91.89	98.35	90.43
HGS	98.26	91.29	97.70	89.85
LSN	97.61	90.75	97.12	89.32

Therefore, the CLAFS-ODLCD methodology is used for improving safety in the digital ecosystem.

## Conclusion

In this study, a CLAFS-ODLCD technique is proposed for securing the digital ecosystem. The CLAFS-ODLCD technique focuses on the classification and recognition of cyberattacks in the IoT infrastructure. To achieve this, the CLAFS-ODLCD approach involves various types of sub-processes namely LSN-based data normalization, CLA-based feature selection subset, SSAE-based classification, and HGS-based hyperparameter tuning. Initially, the CLAFS-ODLCD technique utilizes the LSN approach. In addition, the CLAFS-ODLCD technique employs the CLAFS technique to choose optimum feature subset. Moreover, the detection and classification of cyberattacks are accomplished by using the SSAE technique. Lastly, the HGS optimizer is utilized for optimum hyperparameter selection. The empirical analysis of the CLAFS-ODLCD method is examined under the WSN-DS dataset. The comparison study of the CLAFS-ODLCD method portrayed a superior accuracy value of 99.48% over existing models.

## Data Availability

The data supporting this study’s findings are openly available at https://www.kaggle.com/datasets/bassamkasasbeh1/wsnds, reference number^33^.
